# Molecular docking and network connections of active compounds from the classical herbal formula Ding Chuan Tang

**DOI:** 10.7717/peerj.8685

**Published:** 2020-03-05

**Authors:** Allison Clyne, Liping Yang, Ming Yang, Brian May, Angela Wei Hong Yang

**Affiliations:** 1 Chinese Medicine, School of Health and Biomedical Sciences, RMIT University, Bundoora, VIC, Australia; 2 Department of Pharmacy, Beijing Hospital, Beijing, China; 3 National Center of Gerontology, Institute of Geriatric Medicine, Chinese Academy of Medical Sciences, Beijing, China; 4 Clinical Trial Center, Beijing Hospital, Beijing, China

**Keywords:** Herbal medicine, Complementary medicine, Natural product, Asthma, Respiratory disease, Network pharmacology, Computational analysis

## Abstract

**Background:**

Ding Chuan Tang (DCT), a traditional Chinese herbal formula, has been consistently prescribed for the therapeutic management of wheezing and asthma-related indications since the Song Dynasty (960–1279 AD). This study aimed to identify molecular network pharmacology connections to understand the biological asthma-linked mechanisms of action of DCT and potentially identify novel avenues for asthma drug development.

**Methods:**

Employing molecular docking (AutoDock Vina) and computational analysis (Cytoscape 3.6.0) strategies for DCT compounds permitted examination of docking connections for proteins that were targets of DCT compounds and asthma genes. These identified protein targets were further analyzed to establish and interpret network connections associated with asthma disease pathways.

**Results:**

A total of 396 DCT compounds and 234 asthma genes were identified through database search. Computational molecular docking of DCT compounds identified five proteins (ESR1, KDR, LTA4H, PDE4D and PPARG) mutually targeted by asthma genes and DCT compounds and 155 docking connections associated with cellular pathways involved in the biological mechanisms of asthma.

**Conclusions:**

DCT compounds directly target biological pathways connected with the pathogenesis of asthma including inflammatory and metabolic signaling pathways.

## Introduction

Asthma is defined and classified by its major physical characteristics including airflow obstruction and airway inflammation. Currently the specific cause(s) of asthma remain unknown and curative drugs are not available (Global Initiative for Asthma, 2018).

Historical Chinese medical texts have documented the use of the traditional Chinese herbal formula Ding Chuan Tang (DCT), also known as the Arrest Wheezing Decoction, for the use of asthma-like symptoms such as wheeze and cough since the Song dynasty (960–1279 AD) and the formulation in contemporary use, has been prescribed continually since the Ming Dynasty (1368–1644) (Hoizey & Hoizey, 1993). Contemporary DCT continues to be indicated for wheeze and cough. Modern clinical and experimental evidence suggests its herbal ingredients and their constituent chemical compounds can moderate and manage the symptoms of asthma, including reduction of airway inflammation, hyper-responsiveness and respiratory mucous production, as well as ameliorate damage to the airway epithelium caused by chronic inflammation (Bensky & Barolet, 2009; Chan et al., 2006; Kao et al., 2004). The contemporary DCT formula contains nine herbs: *Ginkgo biloba* L. (Bai Guo), *Ephedra sinica* Stapf (Ma Huang), *Perilla frutescens* (L.) Britton (Su Zi), *Glycyrrhiza uralensis* Fisch (Gan Cao), *Tussilago farfara* L. (Kuan Dong Hua), *Prunus armeniaca* L. (Xing Ren), *Morus alba* L. (Sang Bai Pi), *Scutellaria baicalensis* Georgi (Huang Qin) and *Pinellia ternata* (Thunb.) Makino (Ban Xia) (Bensky & Barolet, 2009; The Plant List, 2013).

As DCT is a multi-herbal formula, identification and analysis of the molecular properties of the chemical compounds could theoretically disclose the biological mechanisms and pathways targeted associated with asthma (Chillistone & Hardman, 2014; Lipinski et al., 2001). Additionally, using a network pharmacology approach through molecular docking potentially identifies DCT-compound target proteins and signaling pathways and how these relate to asthma pathology.

Network pharmacology focuses on the biological networks between ligands (such as DCT-compounds) and disease pathways including target proteins, protein–protein interactions, cellular signaling and biochemical cascades (Shi et al., 2014; Ursa et al., 2011). Computational software was applied to identify potential target proteins for DCT-compounds through the use of pharmacophore models and molecular docking. Molecular docking assists in three-dimensional computer-assisted drug design and within this study, will identify and predict the possible binding sites of DCT-compounds with proteins targeted by asthma genes; thus, identifying the pathological processes in asthma that are directly associated with DCT (Li & Zhang, 2013; Yu & MacKerell, 2017).

## Materials and Methods

The molecular modeling software used included: Discovery Studio 4.5 system from Accelrys Inc. (DS 4.5; San Diego, CA, USA) (BIOVIA, 2015), AutoDock Vina software (Trott & Olson, 2010) and Cytoscape 3.6.0 software (Shannon et al., 2003).

### DCT-compounds identification

**Figure 1:**
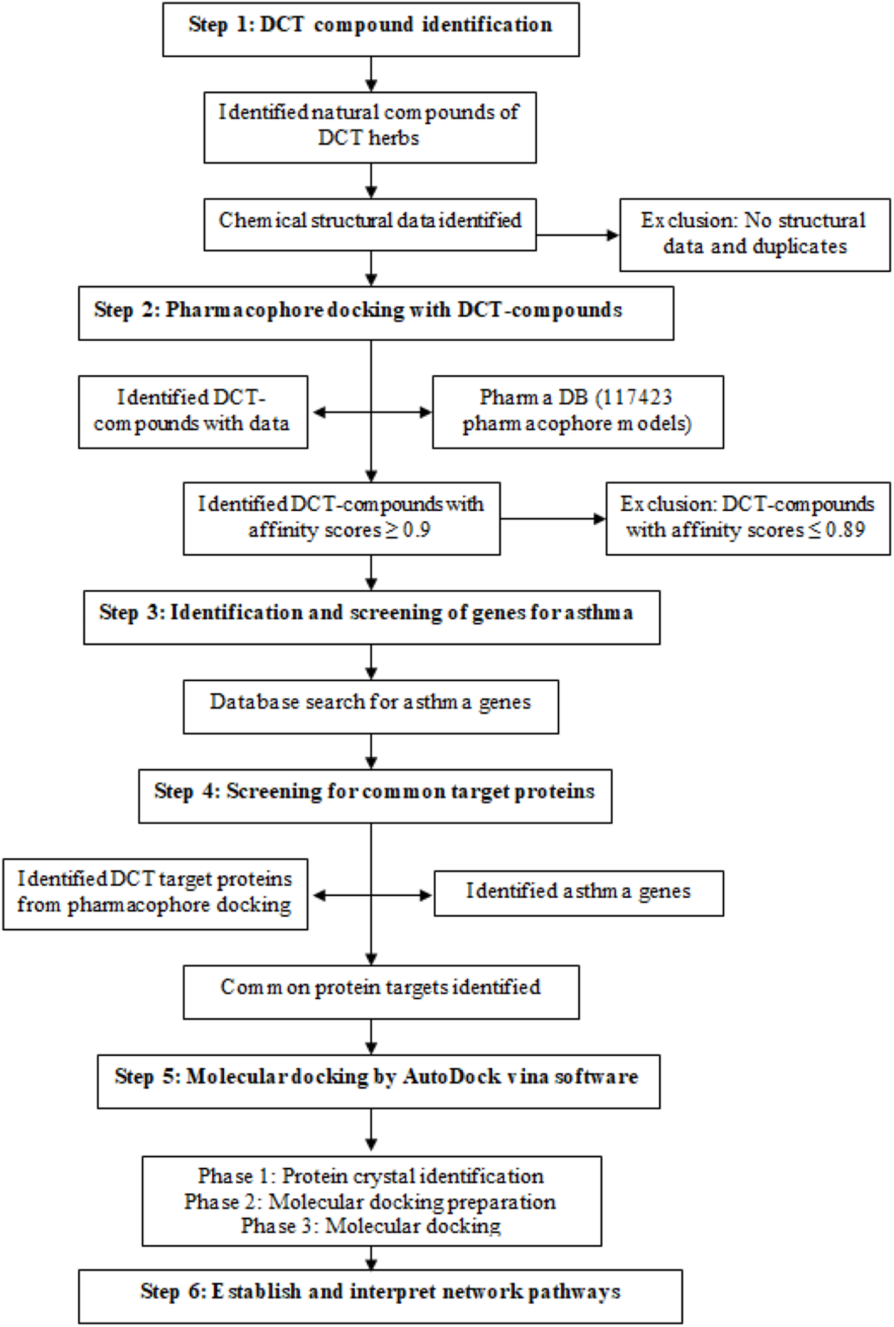
Method flowchart for computational analysis for Ding Chuan Tang.

DCT-compounds were identified by searching the keywords DCT and each of the nine DCT herb names (Chinese, English (common), pharmaceutical and Latin) within five English databases (PubMed, Embase, Science direct, HIT database and CINAHL) and two reference books (*Traditional Chinese medicines: Molecular structures, natural sources and applications* (Zhou, Yan & Xie, 2003), *Chinese herbal medicine: Materia medica* (Bensky, Clavey & Stoger, 2004)). The three-dimensional chemical structures of the DCT-compounds were downloaded from PubChem, and DS 4.5 was used for further optimization (step 1 in Fig. 1).

### Pharmacophore screening with DCT-compounds

Pharmacophore models are constructed from multiple pharmacological features and targets and are used in computational drug discovery to recognize the molecular features of one or more compounds (ligand) required for a lock and key fit with macromolecules (proteins) with the same or similar biological activity (Thangapandian et al., 2011; Wieder et al., 2017). The DS 4.5 software contains a Ligand pharmacophore profiler (LPP) protocol, allowing for virtual screening of multiple chemical compounds against a pharmacophore database (PharmaDB) containing 117,423 pharmacophore models and constructed from 7,028 protein databank (PDB) protein-ligand X-ray crystal structures. This protocol investigates the potential biological actions for the ligands using the pharmacophore models, with the results for the docking within the LPP calculated in fit-values, as a high fit-value indicates the likely comparability of small molecules and target protein binding (Ming, Yi & Yang, 2018). The identified 396 DCT-compounds were virtually screened with the PharmaDB database and the inclusion criteria was set as fit-value 0.9 and above (1.0 fit-value being perfect prediction), to identify the target proteins with the high comparability score (step 2 in Fig. 1).

### Identification and screening of genes for asthma

Genes associated with the asthma disease pathways were identified using “asthma” as a keyword to search the following four databases: Disease gene search engine with evidence (Kim et al., 2013), Genetic Association Database (Becker et al., 2004), Pubmed_Gene (Benson et al., 2013) and GeneCards (Stelzer et al., 2016) (step 3 in Fig. 1). The identified asthma genes were further screened with the pharmacophore and DCT-compound docking results to identify mutual target proteins (step 4 in Fig. 1).

### Molecular docking of DCT-compounds and potential target proteins

DCT-compounds that resulted with fit-value scores ≥0.9 were docked using AutoDock Vina software (Trott & Olson, 2010) with the target proteins identified in the asthma gene and pharmacophore screening (step 5 in Fig. 1). Molecular docking included three phases: (1) crystal complex identification, (2) crystal complex preparation, and (3) molecular docking.

#### Phase 1: crystal complex identification

Docking the DCT-compounds with protein-ligand co-crystallization complexes allows a more specific prediction of binding site locations, docking pose prediction and the likelihood of binding affinity. Phase one of molecular docking required the identification of three-dimensional crystal complex structures of all identified mutual target proteins from RCSB-Protein Data Bank database. For the crystal complexes to be included they must be a human protein, result in an affinity binding score of ≥−9.0 kcal/mol with the identified mutual target proteins, be in the form of a protein-molecule crystal and have a resolution of less than 3 Å.

#### Phase 2: crystal complex preparation

Phase two prepared the identified crystal complexes for docking, with each crystal defined by a configuration (config) file to determine the actions. These config files determined the following for each target protein: the center of the active site (centre_x, centre_y, centre_z), the size of the active site (size_x, size_y, size_z), the maximum number of binding modes to output (num_modes)_ (set to 10) and exhaustiveness (set to 50). During the docking process, a PDBQT file was used in addition to the config file, as the PDBQT file determined the information of potential ligands and receptors required for the target protein docking process.

#### Phase 3: molecular docking

Phase three consisted of AutoDock Vina molecular docking; using Lamarckian genetic algorithm to establish results. The results output as .pdbqt* and log*.txt files and were scored according to virtual docking binding free energy to establish the affinity binding score between the target protein receptors and ligands. For this investigation, affinity binding results ≥−9.0 kcal/mol were selected for the establishment of networks between DCT-compounds and target proteins, as these values suggest strong binding affinity between the DCT-compounds and proteins (Hopkins & Paolini, 2007).

### Establishment of networks

Cytoscape 3.6.0 software (Shannon et al., 2003) was used to establish the networks between docked DCT-compounds and the identified target proteins (step 6 in Fig. 1). The DCT-compounds and target proteins were defined as the nodes of the network, while the affinity binding energy value was the edge of the network. The Network analysis function in Cytoscape 3.6.0 analyzed the features of the network structure through the yfiles plug-in module to draw the network image. This further optimized the layout of the network and identified the most suitable protein targets for DCT-compounds. Kyoto Encyclopedia of Genes and Genomes (KEGG) pathway database (Kanehisa et al., 2017) includes an advanced mapping tool (Search and Color pathway) linked to Cytoscape 3.6.0 software and accessing the affinity binding results from DCT-compounds and protein docking, identified the signaling pathways targeted by DCT.

Data provided from the Protein Data Bank database (PDB) aided in the establishment of network connections between the DCT-compounds and the target proteins post the molecular docking process. Furthermore, the KEGG database (Kanehisa et al., 2017), UniProt database (UniProt Consortium, 2018), Wikipathways (Slenter et al., 2018) and Cell signaling technology pathways (Cell Signaling Technology, 2018) were searched to define and interpret the identified network pathways for the potential mechanisms of action and relevance to the management of asthma.

## Results

### DCT-compound identification

Following the literature search strategies, the original search identified 644 DCT-compounds; however, 109 duplicates and one toxic compound (cyanide) were removed and a further 138 were excluded due to lack of compound identification data such as the molecular structure, molecular formula, simplified molecular-input line-entry system data and physicochemical property data. This resulted in 396 DCT-compounds included for analysis, with 40 from *Ginkgo biloba* L. (Bai Guo), 40 from *Ephedra sinica* Stapf (Ma Huang), 30 from *Perilla frutescens* (L.) Britton (Su Zi), 100 from *Glycyrrhiza uralensis* Fisch (Gan Cao), 15 from *Tussilago farfara* L. (Kuan Dong Hua), 17 from *Prunus armeniaca* L. (Xing Ren), 43 from *Morus alba* L. (Sang Bai Pi), 33 from *Scutellaria baicalensis* Georgi (Huang Qin) and 78 from *Pinellia ternata* (Thunb.) Makino (Ban Xia).

### Pharmacophore screening with DCT-compounds

The virtual screening of the 396 DCT-compounds with the 117,423 pharmacophore models within the PharmaDB resulted in 159 pharmacophore models (potential target proteins). Following this initial hit list, this step resulted in two further sets of outcomes. The first outcome was the reduction of the original hit list of 159 potential target proteins down to 53 due to the inclusion parameter set at fit-value 0.9 and above (Table S1). The second outcome was the reduction of 396 DCT-compounds to 243 DCT-compounds, also due to the 0.9 and above fit-value inclusion parameter (Table S2). This second outcome narrowed down the number of compounds compared during the molecular docking phase to only those DCT-compounds with high affinity binding; as a high fit-value indicates high comparability of small molecules and target protein binding (Ming, Yi & Yang, 2018).

### Identification of genes for asthma

Asthma is classified as a “complex” heritable disease with multiple genes implicated and contributing to its development (Global Initiative for Asthma, 2018). A total of 234 asthma genes were identified, with the results listed in Table S3.

### Screening for common target proteins for DCT target proteins and asthma genes

**Figure 2:**
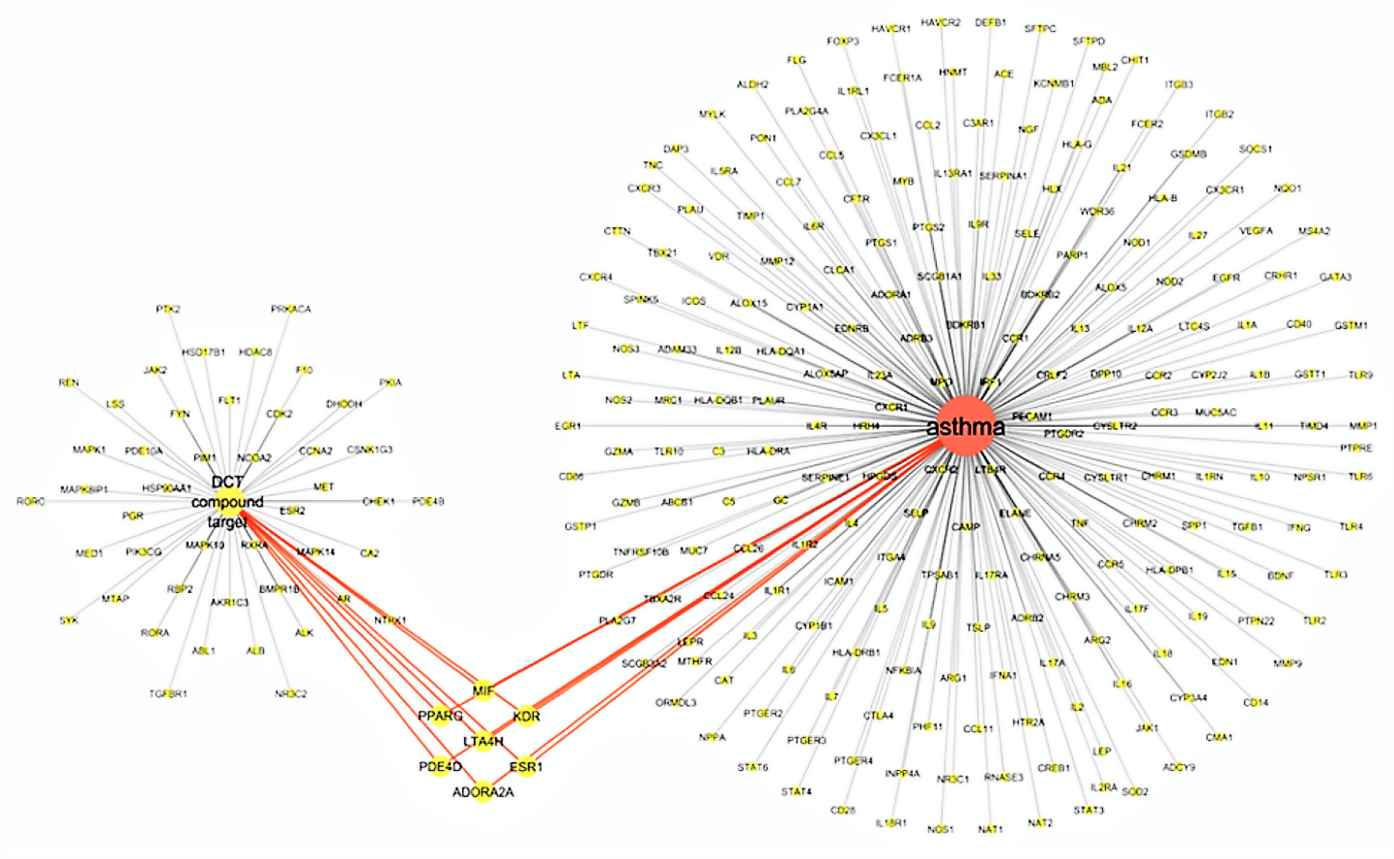
Virtual screening results for Ding Chuan Tang and asthma genes target proteins.

The virtual screening of 53 DCT potential target proteins identified from pharmacophore docking and the 234 identified asthma genes, resulted in seven mutual targets as centrally displayed in Fig. 2, with the list of all proteins provided in Tables S1 and S3. These mutual protein targets included Adenosine A_2A_ receptor (ADORA2A), Estrogen receptors alpha (ESR1), Kinase insert domain receptor (KDR), Leukotriene A4 hydrolase (LTA4H), Macrophage migration inhibitory factor (MIF), cAMP-specific 3′,5′-cyclic phosphodiesterase 4D (PDE4D) and Peroxisome proliferator-activated receptor gamma (PPARG).

In Fig. 2, the group on the left-hand side (Group 1) is the 53 DCT protein targets resulting from pharmacophore virtual screening. Group 2: On the right are all the identified genes associated with asthma. In the center highlighted are the seven proteins both groups target: ADORA2A, ESR1, KDR, LTA4H, MIF, PDE4D and PPARG. At the time of this investigation, the active binding sites, required for Autodock Vina, could not be identified for ADORA2A and MIF; therefore, they were not included in further examination. Five target proteins (ESR1, KDR, LTA4H, PDE4D and PPARG) were included in the next step: Molecular docking with DCT-compounds.

### Molecular docking of active compounds and potential target proteins

#### Phase 1 results: crystal complex identification

Post molecular docking of the five target proteins and the 243 DCT-compounds, a total of 18 human protein-molecule crystal complexes with a resolution of less than 3 Å were identified with affinity scores of ≥−9.0 kcal/mol including three for ESR1 (3erd, 2yja, 2jfa), four for KDR (3vo3, 3vhe, 3cjg, 3cjf), two for LTA4H (4dpr, 3fts), four for PDE4D (1xom, 1tbb, 1tb7, 1zkn), and five for PPARG (5lsg(1), 4jaz, 3sz1_MYR, 3sz1_LU, 3adx) (PDB). The descriptions of the protein-ligand complex and the classifications of these protein crystal complexes as an agonist (promotor or activator) or antagonist (inhibitor or blocker) are defined in Table S4.

#### Phase 2 results: crystal complex preparation

To prepare for molecular docking, the config files for the three-dimensional crystal structures of the five identified target proteins (ESR1, KDR, LTA4H, PDE4D and PPARG) were determined and the parameters for each target protein and corresponding crystals were defined (Table S4).

#### Phase 3 results: molecular docking

The molecular docking results for the specific numbers of compounds from each DCT herb that targeted and docked with affinity scores equal to and over −9.0 kcal/mol to each protein target crystal complex are outlined in Table 1; with many DCT-compounds docking with multiple crystal complexes of the same protein. The three ESR1 crystal complexes resulted in eight docked DCT-compounds from two DCT herbs, *G. uralensis* Fisch (Gan Cao) and *M. alba* L. (Sang Bai Pi) (Table 1). The four KDR crystal complexes resulted in 119 docked DCT-compounds from eight DCT herbs, excluding *P. armeniaca* L. (Xing Ren) (Table 1). The two LTA4H crystal complexes resulted in 139 docked DCT-compounds from all nine DCT herbs, while the four PDE4D crystal complexes resulted in 103 docked DCT-compounds from all nine DCT herbs (Table 1). The five PPARG crystal complexes resulted in 109 docked DCT-compounds from eight DCT herbs, excluding *P. armeniaca* L. (Xing Ren) (Table 1).

**Table 1:** Molecular docking results for Ding Chuan Tang-compounds and target proteins with affinity scores ≥−9.0 kcal/mol.

Target protein Crystal complex (*n* = total compounds docked)	Ding chuan tang herbal ingredient								
	*Ginkgo biloba* L. (Bai Guo)	*Ephedra sinica* Stapf (Ma Huang)	*Perilla frutescens* (L.) Britton (Su Zi)	*Glycyrrhiza uralensis* Fisch (Gan Cao)	*Tussilago farfara* L. (Kuan Dong Hua)	*Prunus armeniaca* L. (Xing Ren)	*Morus alba* L. (Sang Bai Pi)	*Scutellaria baicalensis* Georgi (Huang Qin)	*Pinellia ternata* (Thunb.) Makino (Ban Xia)
ESR1_3erd (*n* = 1)							1		
ESR1_2yja (*n* = 2)				1			1		
ESR1_2jfa (*n* = 6)				4			2		
KDR_3vo3 (*n* = 88)	3	3	8	44	2		20	7	1
KDR_3vhe (*n* = 78)	5	1	7	39	1		14	9	2
KDR_3cjg (*n* = 52)	3	1	3	26	2		14	2	1
KDR_3cjf (*n* = 18)	2		1	6	1		7		1
LTA4H_4dpr (*n* = 122)	10	4	7	59	3	1	21	13	4
LTA4H_3fts (*n* = 54)	7	1	8	22			9	2	5
PDE4D_1xom (*n* = 82)	2		3	48	3	1	17	5	3
PDE4D_1tbb (*n* = 63)	3		3	29	2	1	19	3	3
PDE4D_1tb7 (*n* = 59)	2	1	3	25	1	1	17	7	2
PDE4D_1zkn (*n* = 78)	2		2	45	2	1	18	5	3
PPARG_5slg(1) (*n* = 36)	2		2	12			15	4	1
PPARG_4jaz (*n* = 71)	4	1	6	34	1		17	5	3
PPARG_3sz1_MYR (*n* = 70)	2	1	5	32			13	16	1
PPARG_3sz1_LU (*n* = 69)	2	1	5	32			13	15	1
PPARG_3adx (*n* = 66)	3		5	37	1		14	5	1

**Note:**

ESR1, Estrogen receptors alpha; KDR, Kinase insert domain receptor; LTA4H, Leukotriene A4 hydrolase; PDE4D, cAMP-specific 3’,5’-cyclic phosphodiesterase 4D; PPARG, Peroxisome proliferator-activated receptor gamma.

Affinity binding scores ≥−9.0 kcal/mol resulted with a range between −9 and −13.4 kcal/mol. The range for each target protein is as follows: ESR1 (−9 to −9.9 kcal/mol), KDR (−9 to −11.9 kcal/mol), LTA4H (−9 to −13.4 kcal/mol), PDE4D (−9 to −12 kcal/mol) and PPARG (−9 to −11.8 kcal/mol).

### Establish network connections

**Figure 3:**
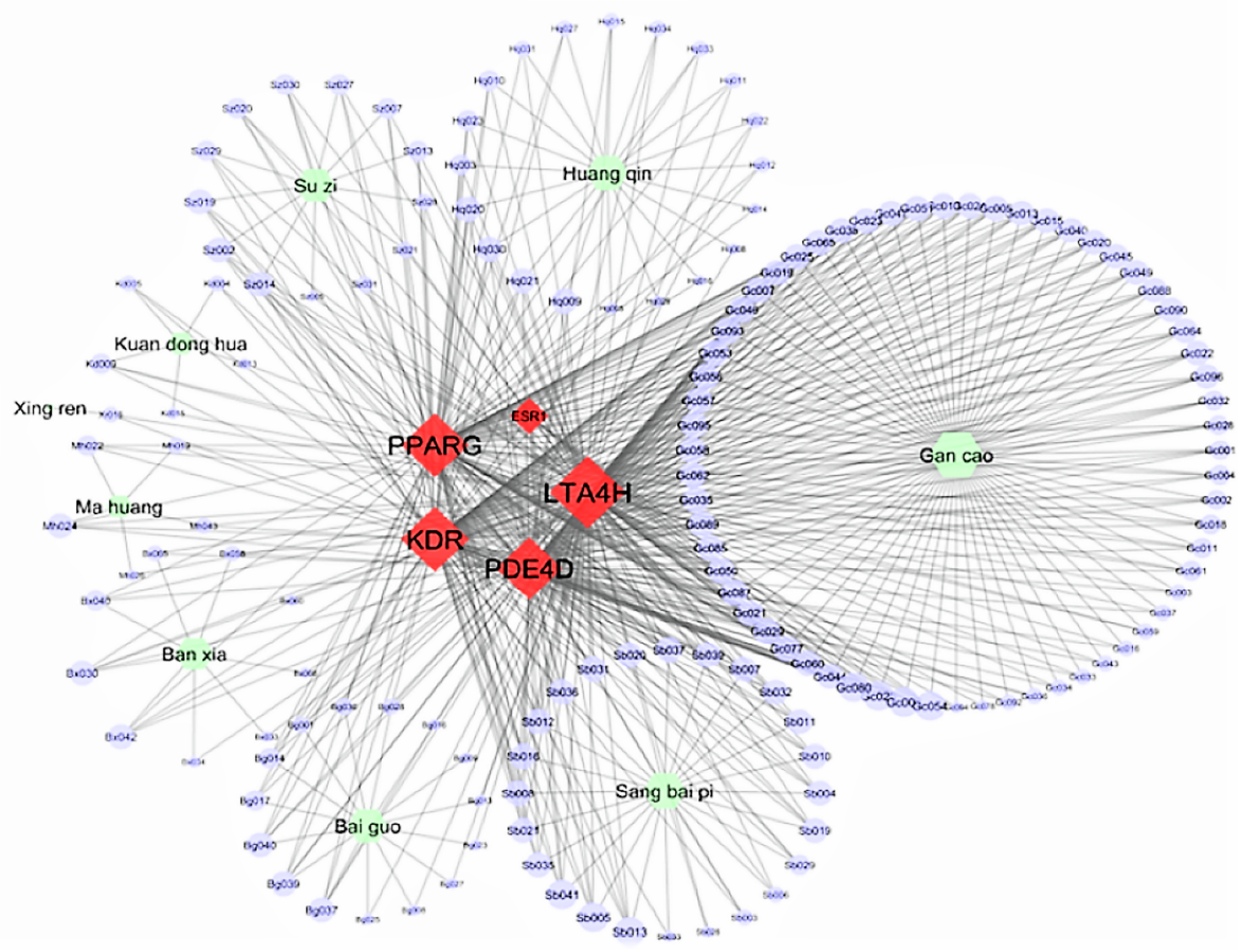
Network connections between Ding Chuan Tang compounds and five target proteins. ESR1, Estrogen receptors alpha; KDR, Kinase insert domain receptor; LTA4H, Leukotriene A4 hydrolase; PDE4D, cAMP-specific 3′,5′-cyclic phosphodiesterase 4D; PPARG, Peroxisome proliferator-activated receptor gamma.

Figure 3 displays the Cytoscape graphic network connections from the molecular docking results between the 243 DCT-compounds and the five target proteins. The Cytoscape 3.6.0 yfiles plug-in module was used to draw and optimize the layout of the network image (Shannon et al., 2003). All five target proteins resulted in 155 network connections with compounds from the nine DCT herbs including: *Ginkgo biloba* L. (Bai Guo) (*n* = 15), *Ephedra sinica* Stapf (Ma Huang) (*n* = 5), *Perilla frutescens* (L.) Britton (Su Zi) (*n* = 13), *Glycyrrhiza uralensis* Fisch (Gan Cao) (*n* = 63), *Tussilago farfara* L. (Kuan Dong Hua) (*n* = 5), *Prunus armeniaca* L. (Xing Ren) (*n* = 1), *Morus alba* L. (Sang Bai Pi) (*n* = 24), *Scutellaria baicalensis* Georgi (Huang Qin) (*n* = 20) and *Pinellia ternata* (Thunb.) Makino (Ban Xia) (*n* = 9) (Fig. 2). Table 2 contains the compound names for each connection node code seen in Fig. 3.

**Table 2:** 155 Target protein connections identified from Ding Chuan Tang compound molecular docking.

Ding Chuan Tang herb	Compound code	Herb chemical compound	Target protein				
			ESR1	KDR	LTA4H	PDE4D	PPARG
*Ginkgo biloba* L. (Bai Guo)	Bg017	catechin		√	√		√
	Bg014	campherol		√	√		√
	Bg040	β-sitosterol		√	√	√	√
	Bg039	stigmasterol		√	√	√	√
	Bg037	sciadopitysin		√	√	√	√
	Bg036	rutin			√		√
	Bg028	isorhamnetine		√	√		
	Bg001	(−)-epicatechin		√	√		
	Bg027	hydroginkgolinic acid			√		
	Bg025	ginkgolide C			√		
	Bg023	ginkgolid B			√		
	Bg016	cardanol			√		
	Bg013	bilobil			√		
	Bg009	anacardic acid B			√		
	Bg008	anacardic acid A			√		
*Ephedra sinica* Stapf (Ma Huang)	Mh026	leucodelphinidin			√		
	Mh019	dobutamine		√	√		
	Mh022	herbacetin		√	√		√
	Mh024	kaempferol rhamnoside		√	√	√	√
	Mh043	tricin		√	√		
*Perilla* *frutescens* (L.) Britton (Su Zi)	Sz020	rosmarinic acid			√	√	√
	Sz013	luteolin		√	√		√
	Sz007	chrysoeriol		√	√		√
	Sz019	phytosterols		√	√	√	√
	Sz014	luteolin-7-O-glucoside		√	√	√	√
	Sz002	apigenin-7-O-glucoside		√	√	√	√
	Sz031	δ-tocopherol			√	√	√
	Sz030	γ-tocopherol		√	√		√
	Sz029	β-tocopherol		√	√		√
	Sz027	α-tocopherol		√		√	
	Sz005	caffeic acid-3-O-glucoside			√		
	Sz028	β-amyrin		√		√	
	Sz021	rosmarinic acid methyl ester		√	√		
*Glycyrrhiza uralensis* Fisch (Gan Cao)	Gc001	11-deoxoglycyrrhetinic		√	√	√	
	Gc002	18β-glycyrrhetinic acid		√	√	√	
	Gc003	2,4,4’-trihydroxychalcone		√	√		
	Gc004	3,3’-dimethylquercetin		√		√	√
	Gc005	3’-methoxyglabridin		√	√	√	√
	Gc006	3-hydroxyglabrol	√	√	√	√	√
	Gc007	4′-O-methylglabridin		√	√	√	√
	Gc010	5-O-methyl licoricidin		√	√	√	√
	Gc011	7-O-methylluteone			√	√	√
	Gc013	apioside		√	√	√	√
	Gc015	dehydroglyasperin C		√	√	√	√
	Gc016	dehydroglyasperin D			√	√	
	Gc018	formononetin		√	√		√
	Gc019	gancaonin A		√	√	√	√
	Gc020	gancaonin B		√	√	√	√
	Gc021	gancaonin C		√	√	√	√
	Gc022	gancaonin D		√	√	√	√
	Gc023	gancaonin E		√	√	√	√
	Gc024	gancaonin F		√	√	√	√
	Gc025	gancaonin p-3′-methylether		√	√	√	√
	Gc026	glabranin	√	√	√	√	√
	Gc028	glabrol		√	√		√
	Gc029	glisoflavanone		√	√	√	√
	Gc032	glycocoumarin		√	√	√	
	Gc033	glycybenzofuran		√	√		
	Gc034	glycyrin			√	√	
	Gc035	glycyrol		√	√	√	√
	Gc036	glycyrrhetic acid acetate		√		√	
	Gc037	glycyrrhetol			√	√	
	Gc038	glycyrrhisoflavanone		√	√	√	√
	Gc040	glycyrrhiza-flavonol A	√	√	√	√	
	Gc043	glyuranolide		√	√		
	Gc044	glyzaglabrin		√	√	√	√
	Gc045	hemileiocarpin		√	√	√	√
	Gc046	hispaglabridin A		√	√	√	√
	Gc047	hispaglabridin B		√	√	√	√
	Gc049	isoglycyrol		√	√	√	√
	Gc050	isolicoflavonol		√	√	√	√
	Gc051	isoliquiritin		√	√	√	√
	Gc053	isoquercitrin		√	√	√	√
	Gc054	isotrifoliol	√	√	√	√	√
	Gc056	kanzonol F		√	√	√	√
	Gc057	kanzonol h		√	√	√	√
	Gc058	licocoumarone		√	√	√	√
	Gc059	licochalcone A			√		√
	Gc060	licocoumarone		√	√	√	√
	Gc061	licofuranocoumarin			√	√	√
	Gc062	licoisoflavone		√	√	√	√
	Gc064	licoleafol		√	√	√	√
	Gc065	licopyranocoumarin		√	√	√	√
	Gc077	licoricidin		√	√	√	√
	Gc078	licoricone			√		
	Gc080	liquiritin	√	√	√	√	√
	Gc085	neoliquiritin		√	√	√	√
	Gc087	ononin		√	√	√	√
	Gc088	paratocarpin B		√	√	√	√
	Gc089	phaseollinisoflavan		√	√	√	√
	Gc090	semilicoisoflavone B		√	√	√	√
	Gc092	uralene				√	√
	Gc093	uralenin		√	√	√	√
	Gc094	uralenneoside			√		
	Gc095	uralenol		√	√	√	√
	Gc096	uralenol-3-methylether		√	√	√	√
*Tussilago farfara* L. (Kuan Dong Hua)	Kd004	bauerenol		√		√	
	Kd005	faradiol		√		√	
	Kd009	kaempferol-3-O-glucoside			√	√	√
	Kd013	senecionine			√		
	Kd015	tussilagonone			√		
*Prunus armeniaca* L. (Xing Ren)	Xr016	prunasine			√	√	
*Morus alba* L. (Sang Bai Pi)	Sb003	α-acetyl-amyrin		√		√	
	Sb004	cyclomorusin		√	√	√	√
	Sb005	cyclomulberrin	√	√	√	√	√
	Sb006	ecdysterone			√	√	
	Sb007	eudraflavone β hydroperoxide		√	√	√	√
	Sb008	kuwanon C		√	√	√	√
	Sb010	kuwanon L		√	√	√	√
	Sb011	kuwanon S		√	√	√	√
	Sb012	kuwanon T		√	√	√	√
	Sb013	leachianone G	√	√	√	√	√
	Sb016	moracin E		√	√	√	√
	Sb019	morocin P		√	√	√	√
	Sb020	morusin		√	√	√	√
	Sb021	mulberrofuran B		√	√	√	√
	Sb026	mulberrofuran N			√		
	Sb029	mulberrofuran Q		√		√	√
	Sb031	mulberroside C		√	√	√	√
	Sb032	oxydihydromorusin		√	√	√	√
	Sb033	oxyresveratrol		√			
	Sb035	sanggenol A		√	√	√	√
	Sb036	sanggenol L		√	√	√	√
	Sb037	sanggenol O		√	√	√	√
	Sb039	sanggenon F	√	√	√	√	√
	Sb041	sanggenon N	√	√	√	√	√
*Scutellaria baicalensis* Georgi (Huang Qin)	Hq003	2′,3′,5,7-tetrahydroxyflavone		√	√		√
	Hq005	3,5,7,2′,6′-pentahydroxy flavanon					√
	Hq008	5,2′-dihydroxy-6,7,8-trimethoxyflav			√		
	Hq009	5,6-dihydroxy-7-O-glucoside-flavone		√	√	√	√
	Hq010	5,7,2′,3′-tetrahydroxyflavone		√	√		√
	Hq011	5,7,2′,6′-tetrahydroxyflavone			√		√
	Hq012	5,7,2′-trihydroxyflavon		√			√
	Hq014	5,8,2′-trihydroxy-7- methoxyflavone					√
	Hq015	5,8-dihydroxy-6,7-dimethoxyflavone		√		√	√
	Hq016	5-hydroxy-7,8-dimethoxyflavone					√
	Hq020	chrysin-7-glucuronide		√	√	√	√
	Hq021	dihydrobaicalin		√	√	√	√
	Hq022	dihydrooroxylin A		√		√	
	Hq023	eriodictyol		√	√		√
	Hq027	oroxylin A		√			√
	Hq029	panicolin					√
	Hq030	scutellarin		√	√	√	√
	Hq031	scutevulin			√		√
	Hq033	wogonin			√		√
	Hq034	wogonoside			√	√	
*Pinellia* *ternata* (Thunb.) Makino (Ban Xia)	Bx030	baicalin		√	√	√	√
	Bx033	bis-(4-hydroxybenzyl) ether			√		
	Bx034	bisabolene			√		
	Bx040	cycloartenol			√	√	√
	Bx042	daucosterol		√	√	√	√
	Bx058	methyl phenanthrene		√		√	
	Bx060	octadecane			√		
	Bx065	sachaliside 1			√	√	
	Bx066	shogaol			√		

**Note:**

Bg, Bai Guo; Bx, Ban Xia; ESR1, Estrogen receptors alpha; Gc, Gan Cao; Hq, Huang Qin; KDR, Kinase insert domain receptor; Kd, Kuan Dong Hua; LTA4H, Leukotriene A4 hydrolase; Mh, Ma Huang; PDE4D, cAMP-specific 3’,5’-cyclic phosphodiesterase 4D; PPARG, Peroxisome proliferator-activated receptor gamma; Sb, Sang Bai Pi; Sz, Su Zi; Xr, Xing Ren.

## Discussion

**Figure 4:**
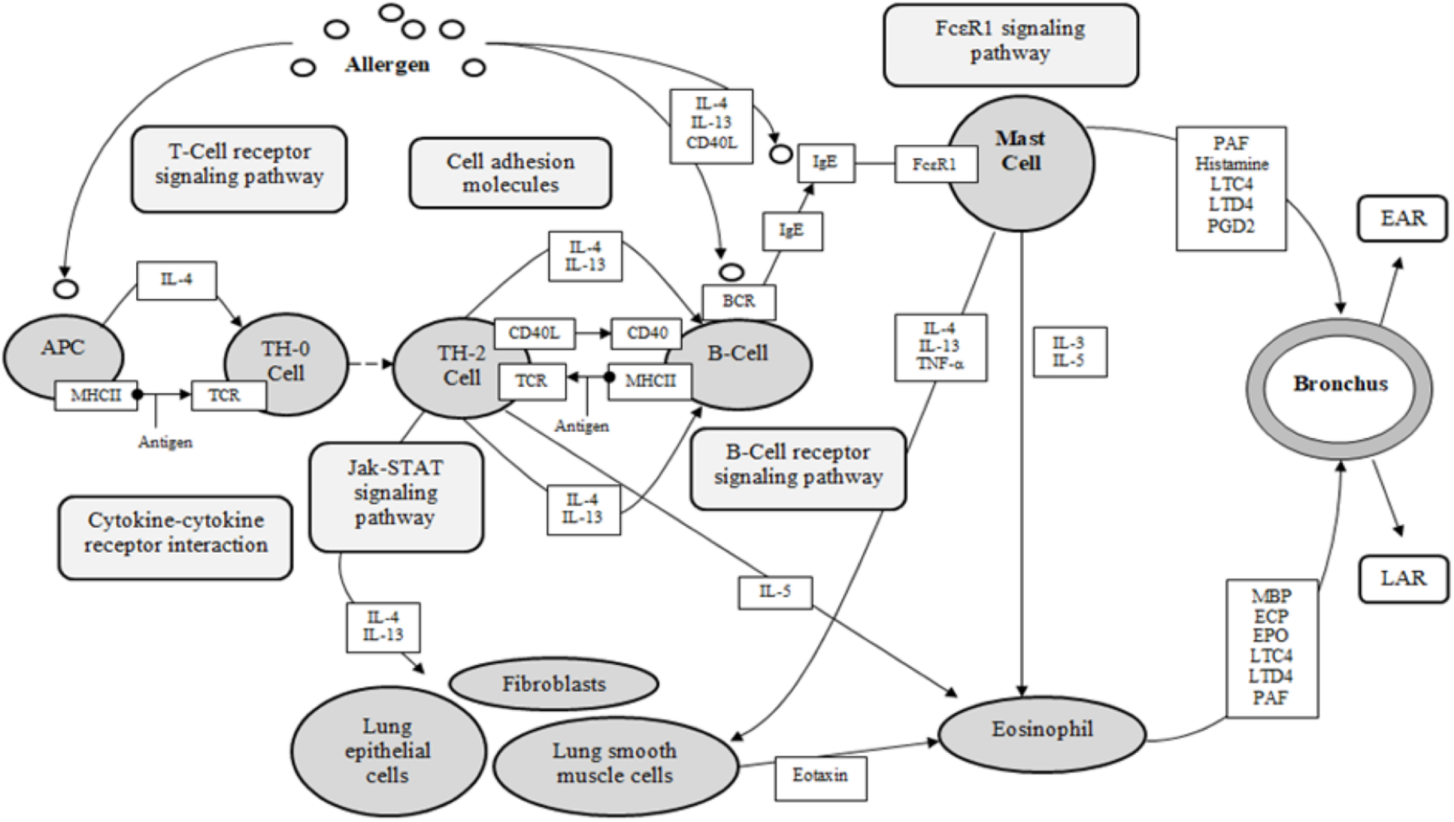
KEGG asthma pathway (adapted from “Asthma pathway- *Homo sapiens*” on KEGG database (Kanehisa et al., 2017)). APC, antigen presenting cell; BCR, B-cell receptor; CD40, cluster of differentiation 40; CD40L, cluster of differentiation 40 L receptor; EAR, early asthmatic response; ECP, eosinophil cationic protein; EPO, eosinophil peroxidase; FcεR1, high affinity immunoglobulin E receptor; IgE, immunoglobulin E; IL-#, Interleukin-#; LAR, late asthmatic response; LTC4, leukotriene C4; LTD4, leukotriene D4; MBP, major basic protein; MHCII, major histocompatibility complexes II; PAF, platelet activating factor; PGD2, Prostaglandin D2; TCR, T-Cell surface receptor; TH-2, T-helper 2 cells; TH-0, naïve T-cells; TNF-α, tumor necrosis factor-α.

Asthma is a complex disease, with multi-cellular interactions, networks and complexes associated and recognized as the foundation of the pathology (KEGG). The five identified target proteins, ESR1, KDR, LTA4H, PDE4D and PPARG, show evidence of involvement in the pathological expression of asthma, with three classified as receptors (ESR1, KDR, PPARG), and two categorized as enzymes (LTA4H, PDE4D) (KEGG, UniProt). These results are significant for the investigation of DCT for asthma, as 155 DCT-compounds resulted in high affinity bonding with these five proteins. Inferences from the molecular docking of DCT-compounds to the target proteins based on the KEGG asthma pathway (Fig. 4) indicate the potential of DCT-compounds to function as agonists (promoting) or antagonists (inhibiting); thus affecting the signaling pathways involved in asthma pathology.

**Figure 5:**
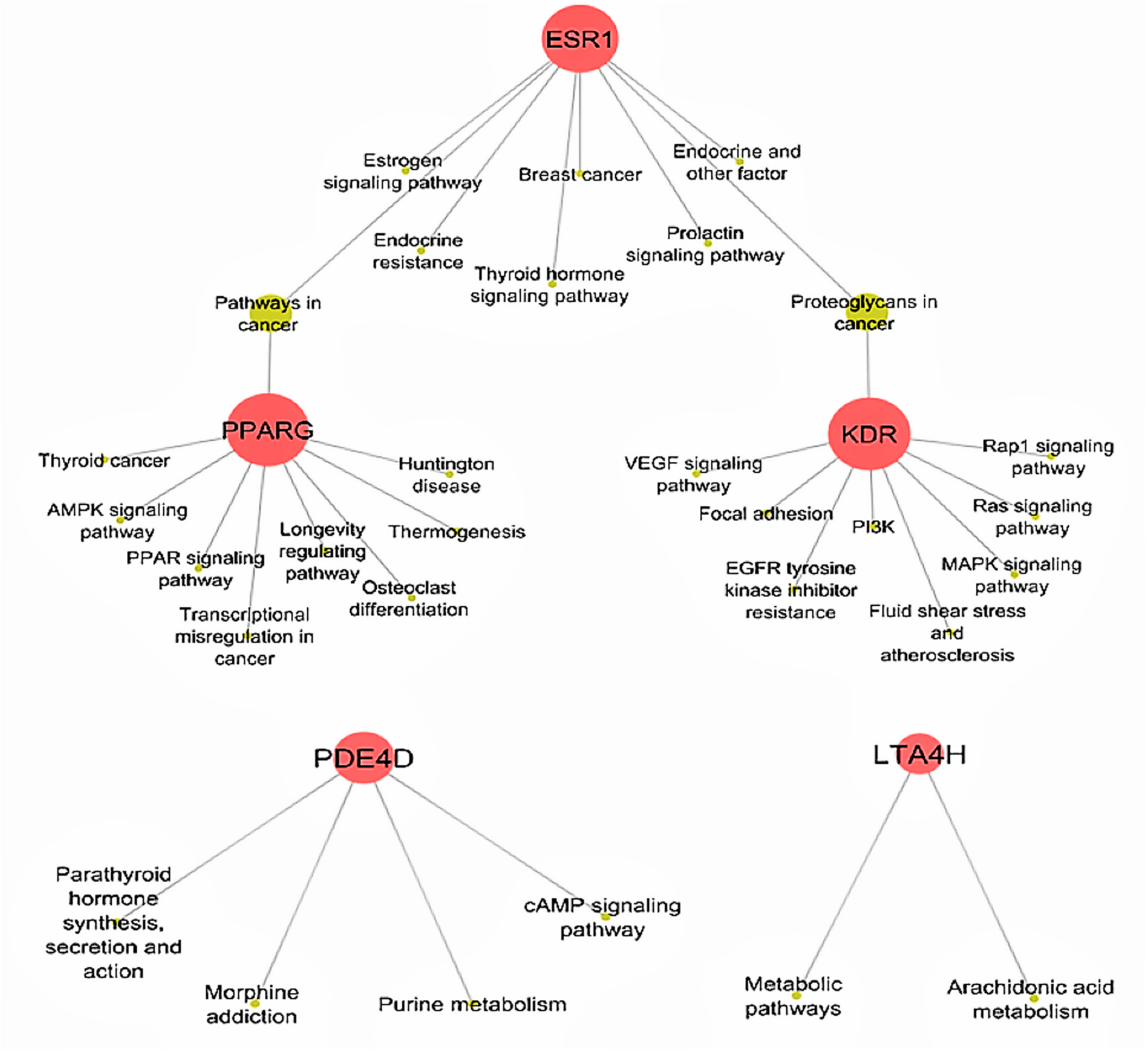
Biological KEGG pathways involved in ESR1, KDR, LTA4H, PDE4D and PPARG. ESR1 (Estrogen receptors alpha) targets eight pathways and shares the Proteoglycans in cancer pathway with KDR (kinase insert domain receptor) and pathways in cancer with PPARG (peroxisome proliferator-activated receptor gamma). All Ding Chuan Tang compounds that target these three proteins potentially increase the synergistic therapeutic outcomes specifically to those pathways. AMPK, 5′ AMP-activated protein kinase; cAMP, cyclic-3′,5′-adenosine monophosphate; EGFR, epidermal growth factor receptor; LTA4H, leukotriene A4 hydrolase; MAPK, mitogen-activated protein kinase; PDE4D, cAMP-specific 3′,5′-cyclic phosphodiesterase 4D; PI3K, phosphatidylinositol 3″-kinase; PPAR, peroxisome proliferator-activated receptor; Rap1, Ras-related protein 1; VEGF, vascular endothelial growth factor.

Determining target protein network connections of all identified DCT-compounds, as opposed to only highlighting the top binding compounds, is important for herbal medicine research, as DCT is prescribed as a multi-herbal formula. Cytoscape 3.6.0 includes applications to associate network connections between the 155 DCT-compounds and target proteins to biological pathways involved in functions such as biological signaling and metabolism through the KEGG database (Shannon et al., 2003). Identifying the biological KEGG pathways targeted by each target protein, as seen in Fig. 5, is the initial step towards understanding the biological activities of the DCT-compounds and how these relate to the disease pathways of asthma. Although some disease pathways are not usually directly associated with asthma, these results suggest there are possible future novel therapeutic approaches for the treatment of asthma to be investigated, as the disease pathways identified all involve the inflammatory process which is the primary pathogenesis of asthma (KEGG; Global Initiative for Asthma, 2018).

### Interpreting network connections

The KEGG database resource is based on current experimental knowledge for “understanding high-level functions and utilities of the biological system” (Kanehisa et al., 2017). The KEGG asthma pathway (Fig. 4) displays the known molecular/cellular interactions and signaling pathways involved in chronic inflammation leading to asthma symptoms. As seen in Fig. 4, there are four signaling pathways involved in the KEGG asthma pathways: (1) T-Cell receptor signaling pathway; (2) B-Cell receptor signaling pathway; (3) the JAK/STAT signaling pathway and (4) the FcεRI signaling pathway. Within this discussion, these pathways will be referred to as the primary pathways. Following on, the pathways further involved within the four primary signaling pathways are referred to as the secondary pathways.

Each of the five identified target proteins, ESR1, KDR, LTA4H, PDE4D and PPARG, have established potential to play significant roles involving both the primary and secondary asthma pathways through many signaling cascade pathways.

### ESR1

Nuclear hormone receptor, ESR1, in respect to the pathology of asthma, is involved in cell proliferation in tissues within the respiratory system, increasing and maintaining the number of cells in the target tissues through an increase in cell division and altering the process of cell death (UniProt). ESR1 participates in two primary KEGG asthma pathways: the B-Cell signaling pathway and the JAK-STAT signaling pathway, through the Prolactin signaling pathway (KEGG). ESR1 acts downstream of both the B-Cell signaling pathway and the JAK-STAT signaling pathway and modulates the immune response through regulation of macrophages, T and B lymphocytes and fibroblasts (KEGG). The Prolactin signaling pathway, targeted by ESR1, activates pathways involved in the inflammatory response including the KEGG secondary MAPK pathway and PI3K pathway and is also involved in the secondary KEGG asthma NF-κB signaling pathway; with ESR1 again acting downstream (KEGG). A protein–protein interaction with ESR1 and NF-κB inhibits NF-κB signaling, resulting in a decrease in the inflammatory response through a reduction of pro-inflammatory cytokines and a dampening of inflammatory gene expression in smooth muscle cells (Kovats, 2015). ESR1 also acts downstream within the Thyroid hormone signaling pathway, involved in the secondary Calcium signaling pathways and the MAPK signaling pathway through moderation of gene transcription and cell proliferation (KEGG). ESR1 participates and acts upstream in the Estrogen signaling pathway involved in the secondary KEGG asthma PI3K-Akt signaling pathway, Calcium signaling pathway and MAPK signaling pathway; targeting immune genes, regulating cell cycles and cell adhesion molecules (KEGG). As ESR1 acts upstream of the Estrogen signaling pathway, there is potential that targeting this gene may inhibit or reduce the effects of those three secondary asthma pathways.

Docking results for ESR1 included two crystal complexes classified as antagonists of ESR1 (2fja and 3erd) and one agonist of ESR1 (2yja) (Table 1). Cyclomulberrin is the single compound binding with multiple ESR1 crystals, suggesting both agonist and antagonist functions. The selective estrogen receptor modulator drug tamoxifen is a known ESR1 agonist and antagonist of ESR1, supporting the findings of cyclomulberrin (Gallo & Kaufman, 1997). The research community recognizes the disparity of inflammatory and anti-inflammatory effects of ESR1, hypothesizing the effects are dependent on the available levels of circulating estrogens or the tissue the ESR1 is present (Yung, Fuseini & Newcomb, 2018; Keselman & Heller, 2015; Dijkstra et al., 2006). Inhibiting ESR1 has been found to (1) reduce airway hyperresponsiveness and thymic development, (2) regulate and decrease pro-inflammatory mediators and (3) hinder the development and advancement of airway changes and remodeling, while promoting ESR1 has been found to impede pro-inflammatory responses in both human and animal studies (Yung, Fuseini & Newcomb, 2018; Keselman & Heller, 2015; Dijkstra et al., 2006).

### KDR

KDR is classified as a type III receptor tyrosine kinase, with alternative names including vascular endothelial growth factor receptor 2 and Fetal Liver Kinase 1. KDR acts as a cell surface receptor for vascular endothelial growth factors A, C and D, all involved in the migration, growth and survival of endothelial cells (Duffy, Bouchier-Hayes & Harmey, 2013; UniProt). KDR is expressed throughout human tissues, acting upstream to the primary KEGG asthma pathway T-cell receptor signaling pathway through the Ras signaling pathway and effects cell growth, survival, cell cycle progression and gene expression (Pate et al. 2010; KEGG). KDR is involved in three of the secondary KEGG asthma pathways including PI3K-Akt signaling pathway, Calcium signaling pathway and MAPK signaling pathway; with the Ras signaling pathway additionally involved in these (KEGG). Two cascade pathways include KDR acting upstream of these three secondary asthma pathways potentially inhibiting the inflammatory outcomes; the VEGF signaling pathway- resulting in cell survival, migration and proliferation, and the RAP1 signaling pathway- involving cell adhesion, migration, proliferation, survival and gene activation (KEGG). KDR acts upstream from MAPK pathway in an additional pathway associated with focal adhesion, with effects including inflammatory cell proliferation and survival (KEGG).

All four KDR crystal complexes are classified as antagonists of KDR functions (KEGG); therefore, the DCT-compounds that docked with all four crystals provide strong evidence as KDR antagonists (Table 1). Evidence supporting the inhibition of KDR as beneficial for asthma involves a variety of pathological pathways including a dampening of the inflammatory asthma response through a reduction in recruitment of pro-inflammatory cells, a decrease circulating cytokines and cell adhesion molecules and the reduction of airway remodeling through smooth muscle hyperplasia (PDB; Uniprot).

### LTA4H

The protein that the human gene LTA4H encodes is an enzyme containing both: (a) hydrolase, with the function of converting leukotriene A4 (LTA4) into leukotriene B4 (LTB4), a proinflammatory mediator and; (b) aminopeptidase, recognized as a biological characteristic of chronic obstructive pulmonary disease (Uniprot; PubMed_Gene). LTB4 promotes the inflammatory response as a mediator of leukocytes including monocytes, eosinophils, macrophages and dendritic cells, along with specific chemotactic function for neutrophils and regulating differentiation of T-cells (Di Gennaro & Haeggstrom, 2014; Liu & Yokomizo, 2015). The primary KEGG asthma FcεRI signaling pathway includes LTA4H through its function in AA metabolism (KEGG). The Eicosanoids pathway instigates both the immune and the inflammatory response through utilizing LTA4H downstream for both the FcεRI signaling pathway and the secondary AA metabolism pathway (KEGG). Pro-inflammatory cells including leukocytes, platelets and fibroblasts, in addition to target tissue of the respiratory system, widely express LTA4H (Uniprot).

Both LTA4H crystal complexes are classified as antagonists of LTA4H, therefore the 38 DCT-compounds that docked with both crystals of LTA4H (Table 1) potentially reduce asthma symptoms such as increased mucous and persistent airway inflammation, through blocking the conversion of LTA4 to LTB4 (PDB; Uniprot).

### PDE4D

Nine isoforms have been identified for the PDE4D gene, each encoding hydrolyzing proteins that break down the second messenger Cyclic adenosine monophosphate (cAMP) (UniProt). The immune effects of cAMP regulate both the innate and adaptive immune responses, with many cells within the respiratory system including leukocytes, macrophages, B-cells and T-cells affected and modulated (Billington et al., 2013; Raker, Becker & Steinbrink, 2016). Increased levels of intracellular cAMP inhibit asthma pathways leading to broncho-constriction through the contraction of respiratory smooth muscle, while as a drug target, it amplifies the anti-inflammatory response and simultaneously inhibits pro-inflammatory immune and cellular factors (Billington et al., 2013; Raker, Becker & Steinbrink, 2016). The secondary KEGG asthma disease pathway PI3K-Akt signaling pathway, involves PDE4D through the cAMP signaling pathway, leading to cell growth, survival and proliferation (KEGG). PDE4D acts upstream of the PI3K-Akt signaling pathway; effectively inhibiting the outcomes and thus reducing inflammatory mediated symptoms (KEGG; Billington et al., 2013).

The four investigated PDE4D crystal complexes have all been shown to be antagonists of PDE4D functions, thus the DCT-compounds docked with them (Table 1) present the potential to reduce bronchoconstriction and reduce the asthma inflammatory cellular mediated symptoms (Billington et al., 2013; Raker, Becker & Steinbrink, 2016; UniProt). PDE4D inhibitors have been used successfully as drug targets for asthma, such as theophylline since the 1930s (Spina, 2008) and Ibudilast, an oral asthma drug approved in Asia (Rolan et al., 2008). These anti-asthma drugs present strong evidence for DCT-compounds as potential future drug candidates through computational demonstration to target PDE4D.

### PPARG

The type II nuclear receptor PPARG is known primarily for the function of regulating the fatty acid pathways and glucose metabolism (UniProt). Asthma development implicates PPARG in regard to the reduction of respiratory inflammation and the permanent remodeling that occurs due to the chronic asthma response (Oh et al., 2009). The activation of PPARG moderates the initiation of pro-inflammatory Th2 cytokines, eosinophils and macrophages, while allowing the expression of anti-inflammatory cellular factors within the lung (Huang & Glass, 2010; Oh et al., 2009). PPARG is involved and acts downstream from the secondary KEGG asthma disease pathway PI3K-Akt signaling pathway through the AMP-activated protein kinase (AMPK) signaling pathway, to inhibit cell growth, cell survival and protein synthesis (KEGG).

All five PPARG crystal complexes are classified as PPARG agonists, suggesting all 108 docked DCT-compounds (Table 1) promote the reduction of pro-inflammatory Th2 cytokines, eosinophils and macrophages, along with lessening asthma chronicity (PDB; Uniprot). Rosiglitazone, a strong PPARG agonist, has shown to significantly improve lung function and decrease IL-5 and IgE in a murine model (El-Naa et al., 2015). Furthermore, a current review of rosiglitazone defines the PPARG agonist as a key regulator of airway inflammation, with strong evidence as an asthma therapeutic target (Banno et al., 2018).

### ADORA2A and MIF

Although these two target proteins were excluded, they are still worth discussion due to virtual screening results. The excluded target protein ADORA2A is a receptor for adenosine, a signaling transductor and second messenger (Uniprot). Adenosine mediates G proteins and in turn activates adenylyl cyclase (an enzyme that synthesizes cAMP), associated with immune function and inflammatory pathways (Uniprot). Adenosine has long been associated with the inhibition of the inflammatory response through the effects on neutrophil and macrophage regulation (Hasko & Cronstein, 2013). The biological processes associated with ADORA2A demonstrate involvement in cell–cell signaling, the cellular defense response and the inflammatory response (Uniprot). Multiple experimental studies have shown agonist ligands of ADORA2A reduce respiratory inflammation (Van Waarde et al., 2018). This research suggests the prospect of the DCT-compounds successfully docked with ADORA2A to display similar agonistic biological effects for asthma.

MIF is a pro-inflammatory cytokine with a regulatory function of the innate immune response (Uniprot). The identified molecular functions of MIF identified and associated to asthma are (1) macrophage mediator and regulator, (2) chemokine attraction and (3) signaling receptor binding (Uniprot). The biological processes of MIF include (1) the inflammatory response, (2) cell proliferation, (3) cell surface receptor signaling pathways, (4) macrophage regulation, (5) leukocyte regulation and (6) B-cell maturation regulation and (7) the innate immune response (Uniprot). Through the direct activation of immune cells and the contribution to airway remodeling in asthma experimental studies (Lan et al., 2018; Hoi, Iskander & Morand, 2007), inhibitors of MIF prove to be a strategic therapeutic target for asthma; thus indicating the potential favorable benefits of the docked DCT-compounds.

### Pharmacological contributions to asthma management

Single pharmaceutical compounds, such as the majority of asthma medications, fundamentally target single molecular mechanisms and as a result may be less efficient at treating complex diseases that have multi-cellular/tissue pathology (Kumar & Zhang, 2018; Aggarwal et al., 2007). It has been proposed that pharmaceuticals targeting molecular signaling pathways are the future for drug discovery as they target multiple protein targets and pathways (Kumar & Zhang, 2018; Aggarwal et al., 2007). The network connection results of this investigation are in line with this theory, as the pharmacological and mechanistic predictions provide evidence, albeit computational, of 155 strong affinity binding connections (≥−9.0 kcal/mol) between DCT-compounds and five proteins associated with multiple asthma KEGG disease pathways (Fig. 3).

Specific DCT-compound insights include the compounds that targeted all crystal complexes of KDR, LTA4H, PDE4D or PPARG (Table 3), as each of the crystal complexes for these proteins are in the same classification; agonist or antagonist. These results suggest that these compounds may promote or inhibit the biological actions associated with each protein through accumulation and synergism. Furthermore, the identified DCT-compounds that bound with all investigated crystal complexes of PDE4D and PPARG are significant justifications for the historical clinical evidence of DCT reducing the symptoms of bronchoconstriction and airway inflammation, as we know these are the outcomes of registered asthma pharmaceutical compounds theophylline (PDE4D antagonist), ibudilast (PDE4D antagonist) and rosiglitazone (PPARG agonist). Within these DCT-compounds there are 12 that bound to all PDE4D crystal complexes and all PPARG crystal complexes and are especially highlighted for further investigation as PDE4D antagonists or PPARG agonists. These included four from *G. uralensis* Fisch (Gan Cao) (apioside-Gc013, gancaonin F-Gc024, paratocarpin B-Gc088 and semilicoisoflavone B-Gc090), five from *M. alba* L. (Sang Bai Pi) (cyclomulberrin-Sb005, kuwanon S-Sb011, moracin E-Sb016, morusin-Sb020 and sanggenon N-Sb041) and three from *S. baicalensis* Georgi (Huang Qin) (chrysin-7-glucuronide-Hq020, dihydrobaicalin-Hq021 and scutellarin-Hq030).

**Table 3:** Ding Chuan Tang-compounds that docked (≥−9.0 kcal/mol) with all investigated crystal complexes.

Target proteins (investigated crystal complexes)	Classification of investigated crystal complexes	DCT-compounds that docked (≥−9.0 kcal/mol) with all investigated crystal complexes
KDR (3vo3; 3vhe; 3cjg; 3cjf)	KDR antagonists	Sciadopitysin-Bg037; gancaonin F-Gc024; hemileiocarpin-Gc045; hispaglabridin B-Gc04; isoglycyrol- Gc049; semilicoisoflavone B-Gc090; uralenin-Gc093; bauerenol- Kd004; cyclomorusin-Sb004; sanggenon N-Sb041; sanggenol O-Sb037
LTA4H (4dpr; 3fts)	LTA4H antagonists	Catechin-Bg017; rutin-Bg036; apigenin-7-O-glucoside-Sz002; rosmarinic acid-Sz020; rosmarinic acid methyl ester-Sz021; dehydroglyasperin C-Gc015; gancaonin B-Gc020; gancaonin C-Gc021; gancaonin D-Gc022; gancaonin F-Gc024; glabranin-Gc026; glisoflavanone-Gc029; glycyrol-Gc035; glycyrrhisoflavanone-Gc038; glycyrrhiza-flavonol A-Gc040; glyzaglabrin-Gc044; hemileiocarpin-Gc04; hispaglabridin A-Gc046; isoglycyrol-Gc049; isoliquiritin-Gc051; isotrifoliol-Gc054; licoleafol-Gc064; ononin-Gc087; paratocarpin B-Gc088; phaseollinisoflavan-Gc089; semilicoisoflavone B-Gc090; kuwanon S-Sb011; moracin E-Sb016; morusin-Sb020; mulberrofuran B-Sb021; mulberroside C-Sb031; sanggenol L-Sb036; sanggenol O-Sb037; sanggenon F-Sb039; sanggenon N-Sb041; scutellarin-Hq030; 5,6-Dihydroxy-7-O-glucoside-flavone-Hq009; baicalin-Bx030
PDE4D (1xom; 1tbb; 1tb7; 1zkn)	PDE4D antagonists	Sciadopitysin-Bg037; stigmasterol-Bg039; luteolin-7-O-glucoside-Sz014; apioside-Gc013; gancaonin E-Gc023; gancaonin F-Gc024; glycyrol-Gc035; glycyrrhisoflavanone-Gc038; glycyrrhiza-flavonol A-Gc040; hemileiocarpin-Gc045; hispaglabridin B-Gc047; hydroxyglabrol-Gc006; isolicoflavonol-Gc050; licocoumarone-Gc058; licoleafol-Gc064; liquiritin-Gc080; paratocarpin B-Gc088; semilicoisoflavone B-Gc090; uralenin-Gc093; 3’-Methoxyglabridin-Gc005; 3-uralene-Gc092; eudraflavone β hydroperoxide-Sb007; cyclomorusin-Sb004; cyclomulberrin-Sb005; kuwanon L-Sb010; kuwanon S-Sb011; moracin E-Sb016; morusin-Sb020; mulberrofuran Q-Sb029; mulberroside C-Sb031; sanggenol L-Sb036; sanggenol O-Sb037; sanggenon N-Sb041; prunasine-Xr016; chrysin-7-glucuronide-Hq020; dihydrobaicalin-Hq021; scutellarin-Hq030
PPARG (5slg(1); 4jaz; 3sz1_MYR; 3sz1_LU; 3adx)	PPARG agonists	Apigenin-7-O-glucoside-Sz002; apioside-Gc013; gancaonin F-Gc024; neoliquiritin-Gc085; paratocarpin B-Gc088,semilicoisoflavone B-Gc090; cyclomulberrin-Sb005; kuwanon S-Sb011; kuwanon T-Sb012; moracin E-Sb016; morusin-Sb020; oxydihydromorusin-Sb032; sanggenol A-Sb035; sanggenon N-Sb041; chrysin-7-glucuronide-Hq020; dihydrobaicalin-Hq021; scutellarin-Hq030; 5,6-Dihydroxy-7-O-glucoside-flavone-Hq009; baicalin-Bx030

**Note:**

Bg, Bai Guo; Bx, Ban Xia; ESR1, Estrogen receptors alpha; Gc, Gan Cao; Hq, Huang Qin; KDR, Kinase insert domain receptor; LTA4H, Leukotriene A4 hydrolase; PDE4D, cAMP-specific 3′,5′-cyclic phosphodiesterase 4D; PPARG, Peroxisome proliferator-activated receptor gamma; Sb, Sang Bai Pi; Sz, Su Zi; Xr, Xing Ren.

## Conclusions

Network pharmacology has facilitated a fundamental, evidence-based platform to determine the biological mechanisms of action for DCT, demonstrated through the multi-asthma protein target capabilities of DCT-compounds. The 155 network docking connections that DCT-compounds formed with proteins also targeted by asthma genes suggest the potential for DCT-compounds to implement therapeutic effects, through inhibition or promotion, to proteins associated with asthma disease pathways and biological networks.

## Supplemental Information

10.7717/peerj.8685/supp-1 Supplemental Information 1 Results for identified Ding Chuan Tang compounds and pharmacophore virtual screening.

10.7717/peerj.8685/supp-2 Supplemental Information 2 Ding Chuan Tang compounds for molecular docking.

10.7717/peerj.8685/supp-3 Supplemental Information 3 Identified asthma genes.

10.7717/peerj.8685/supp-4 Supplemental Information 4 Crystal complex molecular docking target protein parameters.

## Additional Information and Declarations

### Data Availability

The following information was supplied regarding data availability: The raw data are available in the Supplemental Files.
