# Supplementary material for: Molecular docking and network connections of active compounds from the classical herbal formula Ding Chuan Tang
Source: PeerJ. 2020 Mar 5;8:e8685. doi: 10.7717/peerj.8685 (PMC7060917; doi:10.7717/peerj.8685)
Supplement: Supplemental Information 1 [file peerj-08-8685-s001.docx]

**Table S1: Results for identified Ding Chuan Tang compounds and pharmacophore virtual screening**

| Result number | Pharmacophores corresponding to Ding chuan tang target proteins |
| --- | --- |
| N=53 | Adenosine receptor A2a (ADORA2A); Aldo-keto reductase family 1 member C3(AKR1C3); ALK tyrosine kinase receptor (ALK); Androgen receptor (AR); Bone morphogenetic protein receptor type 1B (BMPR1B); cAMP and cAMP-inhibited cGMP 3’,5’-cyclic phosphodiesterase 10A (PDE10A); cAMP-dependent protein kinase catalytic subunit alpha (PRKACA); cAMP-dependent protein kinase inhibitor alpha (PKIA); cAMP-specific 3’,5’-cyclic phosphodiesterase 4B (PDE4B); cAMP-specific 3’,5’-cyclic phosphodiesterase 4D (PDE4D); Carbonic anhydrase 2 (CA2); Casein kinase I isoform gamma-3 (CSNK1G3); C-Jun-amino-terminal kinase-interacting protein 1 (MAPK8IP1); Coagulation factor X (F10); Cyclin-A2 (CCNA2); Cyclin-dependent kinase 2 (CDK2); Dihydroorotate dehydrogenase (quinone) (DHODH); Estradiol 17-beta-dehydrogenase 1 (HSD17B1); Estrogen receptor 1 (ESR1); Estrogen receptor beta (ESR2); Focal adhesion kinase 1 (PTK2); Heat shock protein HSP 90-alpha (HSP90AA); Hepatocyte growth factor receptor (MET); High affinity nerve growth factor receptor (NTRK1); Histone deacetylase 8 (HDAC8); Kinase insert domain receptor (KDR); Lanosterol synthase (LSS); Leukotriene A4 hydrolase (LTA4H); Lysine-specific demethylase 5A (RBP2); Macrophage migration inhibitory factor (MIF); Mediator of RNA polymerase II transcription subunit 1 (MED1); Mineralocorticoid receptor (NR3C2); Mitogen-activated protein kinase 1 (MAPK1); Mitogen-activated protein kinase 10 (MAPK10); Mitogen-activated protein kinase 14 (MAPK14); Nuclear receptor coactivator 2 (NCOA2); Nuclear receptor ROR-alpha (RORA); Nuclear receptor ROR-gamma (RORC); Peroxisome proliferator-activated receptor gamma (PPARG); Phosphatidylinositol-4,5-bisphosphate 3-kinase (PIK3CG); Progesterone receptor (PGR); Renin (REN); Retinoic acid receptor RXR-alpha (RXRA); Serine/threonine-protein kinase Chk 1(CHEK1); Serine/threonine-protein kinase pim-1 (PIM1); Serum albumin (ALB); S-methyl-5”-thioadenosine phosphorylase (MTAP); Spleen tyrosine kinase (SYK); TGF-beta receptor type-1 (TGFBR1); Tyrosine-protein kinase ABL1 (ABL1); Tyrosine-protein kinase Fyn (FYN); Tyrosine-protein kinase JAK2 (JAK2); Vascular endothelial growth factor Receptor 1 (FLT1) |
