# Supplementary material for: Molecular docking and network connections of active compounds from the classical herbal formula Ding Chuan Tang
Source: PeerJ. 2020 Mar 5;8:e8685. doi: 10.7717/peerj.8685 (PMC7060917; doi:10.7717/peerj.8685)
Supplement: Supplemental Information 2 [file peerj-08-8685-s002.docx]

**Table S2: Ding chuan tang compounds for molecular docking**

| **DCT herb** | **DCT compounds** |
| --- | --- |
| Bai guo (n=23) | (-)-Epicatechin; anacardic acid A; anacardic acid B; bilobil; campherol; cardanol; catechin; ceryl alcohol; cis-9, cis-12-Linoleic acid; ginkgolid A; ginkgolid B; ginkgolide C; ginkgolide M; hydroginkgolinic acid; isorhamnetine; nonacasyl alcohol-10; octacosanol-1; quinic acid; riboflavine; rutin; sciadopitysin; stigmasterol; β-Sitosterol |
| Ma huang (n=16) | (-)-Methylephedrine; 1,3,4-Trimethyl-3-cyclohexene-1-carboxaldehyde; 2,4-decadienal; α-linolenic acid; dobutamine; geraniol; herbacetin; kaempferol rhamnoside; lauric acid; leucodelphinidin; methyl palmitate; myrcene; nonanoic acid; n-Triacontanol; trans-2-nonenal; tricin |
| Su zi (n=27) | 2,3-diethyl-5-methylpyrazine; apigenin-7-O-glucoside; arachidic acid; caffeic acid; caffeic acid-3-O-glucoside; chrysoeriol; docosanol; eicosanol; eicosenoic acid; heneicosanol; heptacosanol; luteolin; luteolin-7-O-glucoside; oleic acid; palmitic acid; phytosterols; rosmarinic acid; rosmarinic acid methyl ester; stearic acid; tricosanol; vitamin B (Thiamine); α-tocopherol; β-amyrin; β-tocopherol; γ-tocopherol; δ-tocopherol; Tetracosanol |
| Gan cao (n=67) | 11-Deoxoglycyrrhetinic; 18β-glycyrrhetinic acid; 2,4,4’-Trihydroxychalcone; 3,3’-Dimethylquercetin; 3’-Methoxyglabridin; 3-Hydroxyglabrol; 4’-O-Methylglabridin; 5,6,7,8-Tetrahydro-2,4-dimethylquinoline; 5-O-Methyl licoricidin; 7-O-methylluteone; apioside; dehydroglyasperin C; dehydroglyasperin D; formononetin; gancaonin A; gancaonin B; gancaonin C; gancaonin D; gancaonin E; gancaonin F; gancaonin P-3’-methylether; glabranin; glabrocoumarin; glabrol; glisoflavanone; glycocoumarin; glycybenzofuran; glycyrin; glycyrol; glycyrrhetic acid acetate; glycyrrhetol; glycyrrhisoflavanone; glycyrrhiza-flavonol A; glyuranolide; glyzaglabrin; hemileiocarpin; hispaglabridin A; hispaglabridin B; isoglycyrol; isolicoflavonol; isoliquiritin; isoquercitrin; isotrifoliol; kanzonol F; kanzonol H; licobichalcone; licochalcone A; licocoumarone; licofuranocoumarin; licoisoflavone; licoleafol; licopyranocoumarin; licoricidin; licoricone; liquiritigenin; liquiritin; neoliquiritin; ononin; paratocarpin B; phaseollinisoflavan; semilicoisoflavone B; sinapic acid; uralene; uralenin; uralenneoside; uralenol; uralenol-3-methylether |
| Kuan dong hua (n=7) | Adenosine; bauerenol; faradiol; farfaratin; kaempferol-3-O-glucoside; senecionine; tussilagonone |
| Xing ren (n=7) | citral; ferulic acid; myristic acid; palmitoleic acid; prunasine; γ-Decanolactone; γ-Dodecalactone |
| Sang bai pi (n=23) | cyclomorusin; cyclomulberrin; eudraflavone α hydroperoxide; kuwanon c; kuwanon L; kuwanon S; kuwanon T; leachianone G; moracenin C; morocin P; morusin; mulberrofuran B; mulberrofuran N; mulberrofuran Q; mulberroside C; oxydihydromorusin; oxyresveratrol; sanggenol A; sanggenol O; sanggenon F; sanggenon N; sanggenol L; α-acetyl-amyrin |
| Huang qin (n=25) | 2',3',5,7-tetrahydroxyflavone; 3,5,7,2’,6’-Pentahydroxy flavanonol; 3,5,7,2’,6’-Pentahydroxy flavonol; 5, 8, 2’-Trihydroxy-6,7-dimethoxyflavone; 5,2’-Dihydroxy-6,7,8-trimethoxyflavone; 5,6-Dihydroxy-7-O-glucoside-flavone; 5,7,2',3’-tetrahydroxyflavone; 5,7,2’,6’-tetrahydroxyflavone; 5,7,2’-Trihydroxyflavone; 5,8,2’-Trihydroxy-7- methoxyflavone; 5,8-Dihydroxy-6,7-dimethoxyflavone; 5-Hydroxy-7,8-dimethoxyflavone; 7,2’,6’-Trihydroxy-5-methoxychalcone; chrysin-7-glucuronide; Dihydrobaicalin; dihydrooroxylin A; eriodictyol; neobaicalein; oroxylin A; oroxyloside; panicolin; scutellarin; scutevulin; wogonin; wogonoside |
| Ban Xia (n=48) | 10,13-Eicosadienoic acid; 11-Eicosenoic acid; 1-Octene; 2,5-Dimethyltetradecane; 2,6,10-Trimethyltetradecane; 2-Methyldecane; 2-Methylnonane; 2-Undecanone; 3,4-Dihydroxycinnamyl alcohol; 3-Methyl eicosane; 3-Nonanone; 3-Nonyne; 8-Octadecenoic acid; 9-Heptadecanol; 9-Hexadecenoic acid; 9-Oxo-nonanoic acid; aromandendrene; atractylon; baicalin; bis(4-hydroxybenzyl) ether; bisabolene; cis-4-decenal; citronellal; coniferin; cycloartenol; cytidine; daucosterol; dibutyl phthalate; docosanoic acid; dodecane; ethylpalmitate; farnesane; gingerol; guanosine; Heptadecanoic acid; inosine; isopulegol; methyl phenanthrene; octadecane; pentadecanoic acid; pinellic acid; sachaliside 1; shogaol; thymidine; α-Elemol; α-Monopalmitin; β-Elemene; β-Eudesmol |
