# Supplementary material for: Molecular docking and network connections of active compounds from the classical herbal formula Ding Chuan Tang
Source: PeerJ. 2020 Mar 5;8:e8685. doi: 10.7717/peerj.8685 (PMC7060917; doi:10.7717/peerj.8685)
Supplement: Supplemental Information 3 [file peerj-08-8685-s003.docx]

**Table S3: Identified asthma genes**

| **Number** | **Asthma genes** |
| --- | --- |
| n=234 | Adenosine deaminase (ADA); Adenosine receptor A2a (ADORA2A); Angiotensin-converting enzyme (ACE); Arginase-1 (ARG1); Beta-3 adrenergic receptor (ADRB3); Calcium-activated chloride channel regulator 1 (CLCA1); Calcium-activated potassium channel subunit beta-1 (KCNMB1); C-C motif chemokine 26 (CCL26); CD40 ligand (CD40); Chitotriosidase-1 (CHIT1); Cyclic AMP-responsive element-binding protein 1 (CREB1); Cysteinyl leukotriene receptor 1 (CYSLTR1); Cytochrome P450 2J2 (CYP2J2); Disintegrin and metalloproteinase domain-containing protein 33 (ADAM33); Eosinophil cationic protein (RNASE3); Gasdermin-B (GSDMB); Glutathione S-transferase theta-1 (GSTT1); Granzyme B (GZMB); Hepatitis A virus cellular receptor 1 (HAVCR1); HLA class I histocompatibility antigen, alpha chain G (HLA-G); HLA class II histocompatibility antigen, DP beta 1 chain (HLA-DPB1); HLA class II histocompatibility antigen, DR alpha chain (HLA-DRA); Inactive dipeptidyl peptidase 10 (DPP10); Inducible T-cell costimulator (ICOS); Integrin beta-2 (ITGB2); Interleukin-1 receptor antagonist protein (IL1RN); Interleukin-1 receptor-like 1 (IL1RL1); Interleukin-18 (IL18); Interleukin-18 receptor 1 (IL18R1); Interleukin-33 (IL33); Interleukin-7 receptor subunit alpha (IL7); Lactotransferrin (LTF); Low affinity immunoglobulin epsilon Fc receptor (FCER2); Macrophage mannose receptor 1 (MRC1); Matrix metalloproteinase-9 (MMP9); Multidrug resistance protein 1 (ABCB1); Neuropeptide S receptor (NPSR1); Osteopontin (SPP1); Pulmonary surfactant-associated protein C (SFTPC); Pulmonary surfactant-associated protein D (SFTPD); Receptor-type tyrosine-protein phosphate epsilon (PTPRE); Secretoglobin family 3A member 2 (SCGB3A2); T-cell immunoglobulin and mucin domain-containing protein 4 (TIMD4); T-lymphocyte activation antigen CD86 (CD86); Transcriptional activator Myb (MYB); Tumor necrosis factor receptor superfamily member 10B (TNFRSF10B); Urokinase plasminogen activator surface receptor (PLAUR); 28S ribosomal protein S29 (DAP3); 5-hydroxytryptamine receptor 2A (HTR2A); Adenosine receptor A1 (ADORA1); Adenylate cyclase type 9 (ADCY9); Aldehyde dehydrogenase, mitochondrial (ALDH2); Alpha-1-antitrypsin (SERPINA1); Arachidonate 15-lipoxygenase (ALOX15); Arachidonate 5-lipoxygenase (ALOX5); Arachidonate 5-lipoxygenase-activating protein (ALOX5AP); Arginase-2, mitochondrial (ARG2); Arylamine N-acetyltransferase 1 (NAT1); Arylamine N-acetyltransferase 2 (NAT2); B1 bradykinin receptor (BDKRB1); B2 bradykinin receptor (BDKRB2); Beta-2 adrenergic receptor (ADRB2); Beta-defensin 1 (DEFB1); Beta-nerve growth factor (NGF); Brain-derived neurotrophic factor (BDNF); C3a anaphylatoxin chemotactic receptor (C3AR1); cAMP-specific 3’,5’-cyclic phosphodiesterase 4D (PDE4D); Catalase (CAT); C-C chemokine receptor type 1 (CCR1); C-C chemokine receptor type 2 (CCR2); C-C chemokine receptor type 3 (CCR3); C-C chemokine receptor type 4 (CCR4); C-C chemokine receptor type 5 (CCR5); C-C motif chemokine 2 (CCL2); C-C motif chemokine 24 (CCL24); C-C motif chemokine 5 (CCL5); C-C motif chemokine 7 (CCL7); Chymase (CMA1); Complement C3 (C3); Complement c5 (C5); Corticotropin-releasing factor receptor 1 (CRHR1); CX3C chemokine receptor 1 (CX3CR1); C-X-C chemokine receptor type 1 (CXCR1); C-X-C chemokine receptor type 2 (CXCR2); C-X-C chemokine receptor type 3 (CXCR3); C-X-C chemokine receptor type 4 (CXCR4); Cyclic adenosine monophosphate (CAMP); Cysteinyl leukotriene receptor 2 (CYSLTR2); Cystic fibrosis transmembrane conductance regulator (CFTR); Cytochrome P450 1A1 (CYP1A1); Cytochrome P450 1B1 (CYP1B1); Cytochrome P450 3A4 (CYP3A4); Cytokine receptor-like factor 2 (CRLF2); Cytosolic phospholipase A2 (PLA2G4A); Cytotoxic T-lymphocyte protein 4 (CTLA4); Early growth response protein 1 (EGR1); Endothelin receptor type B (EDNRB); Endothelin-1 (EDN1); Epidermal growth factor receptor (EGFR); E-selectin (SELE); Estrogen receptor 1 (ESR1); Filaggrin (FLG); Forkhead box protein P3 (FOXP3); Fractalkine (CX3CL1); Glucocorticoid receptor (NR3C1); Glutathione S-transferase Mu 1 (GSTM1); Glutathione S-transferase P (GSTP1); Granzyme A (GZMA); H2.0-like homeobox protein (HLX); Hematopoietic prostaglandin D synthase (HPGDS); Hepatitis A virus cellular receptor 2 (HAVCR2); High affinity immunoglobulin epsilon receptor subunit alpha (FCER1A); High affinity immunoglobulin epsilon receptor subunit beta (MS4A2); Histamine H4 receptor (HRH4); Histamine N-methyltransferase (HNMT); HLA class I histocompatibility antigen, B-51 alpha chain (HLA-B); HLA class II histocompatibility antigen, DQ alpha 1 chain (HLA-DQA1); HLA class II histocompatibility antigen, DQ beta 1 chain (HLA-DQB1); HLA class II histocompatibility antigen, DRB1-14 beta chain (HLA-DRB1); Integrin alpha-4 (ITGA4); Integrin beta-3 (ITGB3); Intercellular adhesion molecule 1 (ICAM1); Interferon alpha-1/13 (IFNA1); Interferon gamma (IFNG); Interferon regulatory factor 1 (IRF1); Interleukin-1 alpha (IL1A); Interleukin-1 beta (IL1B); Interleukin-1 receptor type 1 (IL1R1); Interleukin-1 receptor type 2 (IL1R2); Interleukin-10 (IL10); Interleukin-11 (IL11); Interleukin-12 subunit alpha (IL12A); Interleukin-12 subunit beta (IL12B); Interleukin-13 (IL13); Interleukin-13 receptor subunit alpha-1 (IL13RA1); Interleukin-15 (IL15); Interleukin-16 (IL16); Interleukin-17 receptor A (IL17RA); Interleukin-17A (IL17A); Interleukin-17F (IL17F); Interleukin-19 (IL19); Interleukin-2 (IL2); Interleukin-2 receptor subunit alpha (IL2RA); Interleukin-21 (IL21); Interleukin-23 subunit alpha (IL23A); Interleukin-27 (IL27); Interleukin-3 (IL3); Interleukin-4 (IL4); Interleukin-4 receptor subunit alpha (IL4R); Interleukin-5 (IL5); Interleukin-5 receptor subunit alpha (IL5RA); Interleukin-6 (IL6); Interleukin-6 receptor subunit alpha (IL6R); Interleukin-9 (IL9); Interleukin-9 receptor (IL9R); Interstitial collagenase (MMP1); Kinase insert domain receptor (KDR); Lanosterol synthase (LSS); Leptin LEP Eotaxin (CCL11); Leptin receptor (LEPR); Leukotriene A-4 hydrolase (LTA); Leukotriene A4 hydrolase (LTA4H); Leukotriene B4 receptor 1 (LTB4R); Leukotriene C4 synthase (LTC4S); Macrophage metalloelastase (MMP12); Macrophage migration inhibitory factor (MIF); Mannose-binding protein C (MBL2); Metalloproteinase inhibitor 1 (TIMP1); Methylenetetrahydrofolate reductase (MTHFR); Monocyte differentiation antigen CD14 (CD14); Mucin-5AC (MUC5AC); Mucin-7 (MUC7); Muscarinic acetylcholine receptor M1 (CHRM1); Muscarinic acetylcholine receptor M2 (CHRM2); Muscarinic acetylcholine receptor M3 (CHRM3); Myeloperoxidase (MPO); Myosin light chain kinase, smooth muscle (MYLK); NAD(P)H dehydrogenase [quinone] 1 (NQO1); Natriuretic peptides A (NPPA); Neuronal acetylcholine receptor sub-unit 5 (CHRNA5); Neutrophil elastase (ELANE); NF-kappa-B inhibitor alpha (NFKBIA); Nitric oxide synthase, brain (NOS1); Nitric oxide synthase, endothelial (NOS3); Nitric oxide synthase, inducible (NOS2); Nucleotide-binding oligomerization domain-containing protein 1 (NOD1); Nucleotide-binding oligomerization domain-containing protein 2 (NOD2); ORM1-like protein 3 (ORMDL3); Peroxisome proliferator-activated receptor gamma (PPARG); PHD finger protein 11 (PHF11); Plasminogen activator inhibitor 1 (SERPINE1); Platelet endothelial cell adhesion molecule (PECAM1); Platelet-activating factor acetylhydrolase (PLA2G7); Poly [ADP-ribose] polymerase 1 (PARP1); Prostaglandin D2 receptor 2 (PTGDR); Prostaglandin D2 receptor 2 (PTGDR2); Prostaglandin E2 receptor EP3 subtype (PTGER3); Prostaglandin E2 receptor EP4 subtype (PTGER2); Prostaglandin E2 receptor EP4 subtype (PTGER4); Prostaglandin G/H synthase 1 (PTGS1); Prostaglandin G/H synthase 2 (PTGS2); P-selectin (SELP); Serine protease inhibitor Kazal-type 5 (SPINK5); Serum paraoxonase/arylesterase 1 (PON1); Signal transducer and activator of transcription 3 (STAT3); Signal transducer and activator of transcription 4 (STAT4); Signal transducer and activator of transcription 6 (STAT6); Src substrate cortactin (CTTN); Superoxide dismutase [Mn] (SOD2); Suppressor of cytokine signaling 1 (SOCS1); T-box transcription factor (TBX21); T-cell-specific surface glycoprotein CD28 (CD28); Tenascin (TNC); Thromboxane A2 receptor (TBXA2R); Thymic stromal lymphopoietin (TSLP); Toll-like receptor 10 (TLR10); Toll-like receptor 2 (TLR2); Toll-like receptor 3 (TLR3); Toll-like receptor 4 (TLR4); Toll-like receptor 6 (TLR6); Toll-like receptor 9 (TLR9); Trans-acting T-cell-specific transcription factor GATA-3 (GATA3); Transforming growth factor beta-1 (TGFB1); Tryptase alpha/beta-1 (TPSAB1); Tumor necrosis factor (TNF); Type I inositol 3,4-bisphosphate 4-phosphatase (INPP4A); Tyrosine-protein kinase JAK1 (JAK1); Tyrosine-protein phosphatase non-receptor type 22 (PTPN22); Urokinase-type plasminogen activator (PLAU); Uteroglobin (SCGB1A1); Vascular endothelial growth factor A (VEGFA); Vitamin D3 receptor (VDR); Vitamin D-binding protein (GC); WD repeat-containing protein 36 (WDR36) |
