# Supplementary material for: Molecular docking and network connections of active compounds from the classical herbal formula Ding Chuan Tang
Source: PeerJ. 2020 Mar 5;8:e8685. doi: 10.7717/peerj.8685 (PMC7060917; doi:10.7717/peerj.8685)
Supplement: Supplemental Information 4 [file peerj-08-8685-s004.docx]

**Table S4: Crystal complex molecular docking target protein parameters**

| **Protein crystal complexes** | **Protein-ligand crystal complex description** | **Protein agonist or antagonist** | **Docking parameters** |
| --- | --- | --- | --- |
| **ESR1** | | | |
| **3erd** | ESR1 in complex with PDB ligand Diethylstilbestrol | ESR1 antagonist | The center of the active site of protein crystal ESR1-3erd was set as center_x_= 5.3, center_y_= -0.2, center_z_= -5.5; the size of the active center was set as size_x_= 17.0, size_y_= 17.0, size_z_= 17.0; the parameters were num_modes_=10, exhaustiveness=50; others were set as default. |
| **2yja** | ESR1 in complex with PDB ligand Estradial | ESR1 agonist | The center of the active site of protein crystal ESR1-2yja was set as center_x_= 23.7, center_y_= 11.1, center_z_= 10.2; the size of the active center was set as size_x_= 21.0, size_y_= 21.0, size_z_= 21.0; the parameters were num_modes_=10, exhaustiveness=50; others were set as default. |
| **2jfa** | ESR1 in complex with PDB ligand Raloxifene | ESR1 antagonist | The center of the active site of protein crystal ESR1-2jfa was set as center_x_= -44.3, center_y_= 13.1, center_z_= 3.5; the size of the active center was set as size_x_= 27.0, size_y_= 27.0, size_z_= 27.0; the parameters were num_modes_=10, exhaustiveness=50; others were set as default. |
| **KDR** | | | |
| **3vo3** | KDR in complex with PDB ligand N-[3-({2-[(cyclopropylcarbonyl)amino]imidazo[1,2-b]pyridazin-6-yl}oxy)phenyl]-1,3-dimethyl-1H-pyrazole-5-carboxamide | KDR antagonist | The center of the active site of protein crystal KDR-3vo3 was set as center_x_= 25.6, center_y_= -27.7, center_z_= -13.2; the size of the active center was set as size_x_= 31.5, size_y_= 31.5, size_z_= 31.5; the parameters were num_modes_=10, exhaustiveness=50; others were set as default. |
| **3vhe** | KDR in complex with PDB ligand 1-{2-fluoro-4-[(5-methyl-5H-pyrrolo[3,2-d]pyrimidin-4-yl)oxy]phenyl}-3-[3-(trifluoromethyl)phenyl]urea | KDR antagonist | The center of the active site of protein crystal KDR-3vhe was set as center_x_= -24.9, center_y_= -1.1, center_z_= -10.5; the size of the active center was set as size_x_= 22.7, size_y_= 22.7, size_z_= 22.7; the parameters were num_modes_=10, exhaustiveness=50; others were set as default. |
| **3cjg** | KDR in complex with PDB ligand N~4~-methyl-N~4~-(3-methyl-1H-indazol-6-yl)-N~2~-(3,4,5-trimethoxyphenyl) pyrimidine-2,4-diamine | KDR antagonist | The center of the active site of protein crystal KDR-3cjg was set as center_x_= 8.2, center_y_= 40.8, center_z_= 7.4; the size of the active center was set as size_x_= 28.0, size_y_= 28.0, size_z_= 28.0; the parameters were num_modes_=10, exhaustiveness=50; others were set as default. |
| **3cjf** | KDR in complex with PDB ligand N~4~-(3-methyl-1H-indazol-6-yl)-N~2~-(3,4,5-trimethoxyphenyl)pyrimidine-2,4-diamine | KDR antagonist | The center of the active site of protein crystal KDR-3cjf was set as center_x_= 18.4, center_y_= 17.7, center_z_= 18.9; the size of the active center was set as size_x_= 18.1, size_y_= 18.1, size_z_= 18.1; the parameters were num_modes_=10, exhaustiveness=50; others were set as default. |
| **LTA4H** | | | |
| **4dpr** | LTA4H in complex with PDB ligand L-Captopril | LTA4H antagonist | The center of the active site of protein crystal LTA4H-4dpr was set as center_x_= 4.5, center_y_= -8.1, center_z_= 1.1; the size of the active center was set as size_x_= 16.0, size_y_= 16.0, size_z_= 16.0; the parameters were num_modes_=10, exhaustiveness=50; others were set as default. |
| **3fts** | LTA4H in complex with PDB ligand Resveratrol | LTA4H antagonist | The center of the active site of protein crystal LTA4H-3fts and PDB ligand Resveratrol was set as center_x_= -25.2, center_y_= 0.9, center_z_= -2.2; the size of the active center was set as size_x_= 21.0, size_y_= 21.0, size_z_= 21.0; the parameters were num_modes_=10, exhaustiveness=50; others were set as default. |
| **PDE4D** | | | |
| **1xom** | PDE4D in complex with PDB ligand Cilomilast | PDE4D antagonist | The center of the active site of protein crystal PDE4D-1oxm was set as center_x_= 14.0, center_y_= 6.0, center_z_= 13.2; the size of the active center was set as size_x_= 27.0, size_y_= 27.0, size_z_= 27.0; the parameters were num_modes_=10, exhaustiveness=50; others were set as default. |
| **1tbb** | PDE4D in complex with PDB ligand Rolipram | PDE4D antagonist | The center of the active site of protein crystal PDE4D-1tbb and PDB ligand Rolipram was set as center_x_= 14.2, center_y_= 6.1, center_z_= 11.8; the size of the active center was set as size_x_= 28.0, size_y_= 28.0, size_z_= 28.0; the parameters were num_modes_=10, exhaustiveness=50; others were set as default. |
| **1tb7** | PDE4D in complex with PDB ligand Adenosine monophosphate | PDE4D antagonist | The center of the active site of protein crystal PDE4D-1tb7 was set as center_x_= 17.8, center_y_= 5.7, center_z_= 68.2; the size of the active center was set as size_x_= 21.0, size_y_= 21.0, size_z_= 21.0; the parameters were num_modes_=10, exhaustiveness=50; others were set as default. |
| **1zkn** | PDE4D in complex with PDB ligand 3-Isobutyl-1-Methylxanthine | PDE4D antagonist | The center of the active site of protein crystal PDE4D-1zkn was set as center_x_= 21.0, center_y_= -5.1, center_z_= 29.7; the size of the active center was set as size_x_= 27.0, size_y_= 27.0, size_z_= 27.0; the parameters were num_modes_=10, exhaustiveness=50; others were set as default. |
| **PPARG** | | | |
| **5lsg** | PPARG in complex with PDB ligand Betulinic acid | PPARG agonist | The center of the active site of protein crystal PPARG-5lsg was set as center_x_= 18.2, center_y_= 17.8, center_z_= 14.5; the size of the active center was set as size_x_= 21.0, size_y_= 21.0, size_z_= 21.0; the parameters were num_modes_=10, exhaustiveness=50; others were set as default. |
| **4jaz** | PPARG in complex with PDB ligand Resveratrol | PPARG agonist | The center of the active site of protein crystal PPARG-4jaz was set as center_x_= 16.6, center_y_= 23.2, center_z_= 8.2; the size of the active center was set as size_x_= 21.0, size_y_= 21.0, size_z_= 21.0; the parameters were num_modes_= 10, exhaustiveness= 50; others were set as default. |
| **3sz1_MYR** | PPARG in complex with PDB ligand Myristic acid | PPARG agonist | The center of the active site of protein crystal PPARG-3sz1_MYR was set as center_x_= 35.6, center_y_= -19.9, center_z_= 38.4; the size of the active center was set as size_x_= 25.5, size_y_= 25.5, size_z_= 25.5; the parameters were num_modes_=10, exhaustiveness=50; others were set as default. |
| **3sz1_LU** | PPARG in complex with PDB ligand Lutetium | PPARG agonist | The center of the active site of protein crystal PPARG-3sz1_LU was set as center_x_= 39.7, center_y_= -23.1, center_z_= 42.6; the size of the active center was set as size_x_= 18.3, size_y_= 18.3, size_z_= 18.3; the parameters were num_modes_=10, exhaustiveness=50; others were set as default. |
| **3adx** | PPARG in complex with PDB ligand Indomethacin | PPARG agonist | The center of the active site of protein crystal PPARG-3adx was set as center_x_= 21.3, center_y_= 65.9, center_z_= 16.1; the size of the active center was set as size_x_= 27.0, size_y_= 27.0, size_z_= 27.0; the parameters were num_modes_=10, exhaustiveness=50; others were set as default. |
| Abbreviations: ESR1: Estrogen receptors alpha; KDR: Kinase insert domain receptor; LTA4H: Leukotriene A4 hydrolase; PDB: Protein Databank; PDE4D: cAMP-specific 3',5'-cyclic phosphodiesterase 4D; PPARG: Peroxisome proliferator-activated receptor gamma. | | | |
